# Long-term effects of SARS-CoV-2 infection in hospitalized children: findings from an Italian single-center study

**DOI:** 10.1186/s13052-024-01596-y

**Published:** 2024-02-14

**Authors:** Valeria Calcaterra, Veronica Maria Tagi, Enza D’Auria, Alessia Lai, Sara Zanelli, Chiara Montanari, Elia Maria Biganzoli, Giuseppe Marano, Elisa Borghi, Valentina Massa, Agostino Riva, Gianvincenzo Zuccotti

**Affiliations:** 1 Department of Internal Medicine and Therapeutics, University of Pavia, Via Aselli 2, 27100 Pavia, Italy; 2 Pediatric Department, Buzzi Children’s Hospital, 20154 Milano, Italy; 3 Department of Biomedical and Clinical Sciences, University of Milan, 20157 Milan, Italy; 4 Department of Health Sciences, University of Milan, 20142 Milan, Italy; 5 III Division of Infectious Diseases, ASST Fatebenefratelli Sacco, Luigi Sacco Hospital, 20157 Milan, Italy

**Keywords:** SARS-CoV-2, Infection, COVID, Long COVID, Long-term effects, Hospitalized, Children

## Abstract

**Background:**

Limited evidence exists regarding the association between COVID-19 and Long COVID manifestations in children, particularly concerning variants of concern (VOCs). We aimed to characterize a cohort of pediatric patients hospitalized with confirmed acute SARS-CoV-2 and monitor them for Long COVID symptoms. Additionally, it seeks to explore any potential correlations between VOCs and clinical symptoms.

**Methods:**

We conducted a prospective study involving children hospitalized from November 2021 to March 2023, with confirmed acute SARS-CoV-2 infection. A telephone survey was conducted at 3-6-12 months after discharge.

**Results:**

We included 167 patients (77 F/90 M). Upon hospital admission, 95.5% of patients presented as symptomatic. Regarding patients for whom it was feasible to determine the SARS-CoV-2 variant (*n* = 51), the Delta variant was identified in 11 children (21.6%) and Omicron variant in the remaining 40 patients (78.4%: 27.5% BA.1 variant; 15% BA.2 variant; 57.5% BA.5 variant). 19 patients (16.5%) reported experiencing at least one symptom indicative of Long COVID (weight loss 31.6%, inappetence 26.3%, chronic cough 21.1%, fatigue 21.1%, and sleep disturbances, wheezing, abdominal pain and mood disorders 15.8%). In only 4 patients with Long COVID we could identified a specific SARS-CoV-2 variant (3 Omicron: 2 BA.1 and 1 BA.2; 1 Delta).

**Conclusions:**

this study underscores that long COVID is a significant concern in the pediatric population. Our data reinforce the importance of continuously monitoring the impact of long-COVID in infants, children, and adolescents. A follow-up following SARS-CoV-2 infection is therefore advisable, with symptom investigation tailored to the patient’s age.

**Supplementary Information:**

The online version contains supplementary material available at 10.1186/s13052-024-01596-y.

## Background

Following the emergence of the COVID-19 pandemic, a notable increase in persistent symptoms has been observed in individuals previously infected with SARS-CoV-2, in contrast to those who have not encountered the virus. The term “Long COVID” is used to describe the continuation or development of these symptoms for at least two months, occurring three months after the initial SARS-CoV-2 infection, with no other plausible explanation [1]. Long COVID affects survivors of COVID-19 across the spectrum of disease severity, including mild-to-moderate cases that did not require respiratory support or hospitalization [2]. Furthermore, Long COVID symptoms have been reported in children and adolescents, including those who experienced asymptomatic or mildly symptomatic COVID-19. It is worth noting that similar post-viral symptoms have been observed in the context of prior human coronavirus diseases, such as Middle East respiratory syndrome (MERS) and severe acute respiratory syndrome (SARS) [3].

Radiological and respiratory function studies have indicated that pulmonary scarring might be a common consequence of COVID-19, potentially explaining the persistent dyspnea and cough seen in Long COVID [4]. Long-term pulmonary dysfunction has been identified in children who have recovered from COVID-19, underscoring the importance of understanding these complications [5]. Additionally, structural and metabolic abnormalities in the brain have been reported among individuals with prior SARS-CoV-2 infection, even in cases with mild symptomatology, possibly attributed to persistent neuroinflammatory processes [6], supported by the finding of SARS-CoV-2 genes and proteins and pathological immune and vascular activations in the brainstem of deceased COVID-19 victims [7]. Radiological evidence of cardiac injury in Long COVID exists, with uncertain long-term implications, yet it may elucidate the persistence of chest pain, heart palpitations, and tachycardia for up to six months post-recovery [2]. In addition to pulmonary, neurological, and cardiac involvement, radiological damage and functional impairment of other organs, such as the liver, spleen, and kidneys, have been reported, persisting for at least 2–3 months after hospital discharge [8, 9].

While the precise mechanisms behind Long COVID remain elusive, several hypotheses regarding its pathophysiology have been proposed [10]. Dysfunction of T-cells might contribute to Long COVID development through autoimmune processes [11], wherein SARS-CoV-2 induces antigen-presenting cells to present antigens to autoreactive T-cells [12]. B-cells may also play a role by producing antiphospholipid autoantibodies detected in 52% of patients in association with neutrophil hyperactivity, and is often associated with more severe clinical outcomes [13]. Furthermore, autoantibodies against interferons, neutrophils, connective tissues, cyclic citrullinated peptides, and cell nuclei have been identified in 10–50% of COVID-19 patients [14]. These autoantibodies, reminiscent of those found in chronic autoimmune diseases like lupus erythematosus and rheumatoid arthritis [15], may contribute to Long COVID symptoms, including fatigue, joint pain, concentration difficulties, and headaches [16–17]. Other potential contributors to Long COVID pathophysiology in both children and adults, include residual inflammation following SARS-CoV-2 multisystem inflammatory syndrome (MIS), characterized by lymphopenia and elevated pro-inflammatory markers [18], as well as deep alterations in the gut microbiome, which have been observed up to 30 days after disease resolution and correlated with prolonged SARS-CoV-2 shedding [19].

Despite these insights, limited evidence exists regarding the association between COVID-19 and Long COVID manifestations in children, particularly concerning variants of concern (VOCs). Studies have shown that certain VOCs, such as the Delta variant, exhibit significantly higher viral loads in the upper respiratory tract of adults, raising questions about their impact on children [20]. Another study suggests that seizures may be a frequent and early sign of the Omicron variant in children with acute infections [21].

This study aims to clinically characterize a cohort of pediatric patients who were hospitalized due to SARS-CoV-2 infection and monitor them for Long COVID symptoms following discharge. Additionally, it seeks to explore any potential correlations between variants of concern (VOCs) and clinical symptoms, where relevant.

## Patients and methods

### Patients

**Fig. 1:**
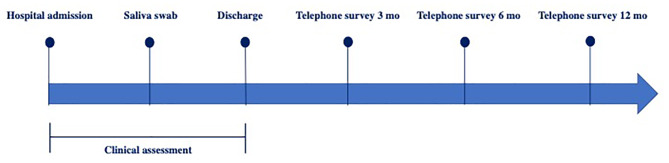
Flowchart illustrating the monitoring process

We conducted a prospective study involving children hospitalized at Milan’s Buzzi Children’s Hospital in Italy from November 1st, 2021, to March 30th, 2023, with confirmed acute SARS-CoV-2 infection as documented by nasal swab upon admission. During hospitalization, written consent was obtained from both the child and their accompanying parent, allowing for the collection of saliva swabs to determine SARS-CoV-2 variants. Furthermore, the patients were asked to participate in long-term monitoring after discharge, and those with at least six months of follow-up were included in the analysis. A comprehensive clinical assessment was conducted during hospitalization, collecting data on demographics, symptoms, and comorbidities in the acute phase. To assess the development of Long COVID symptoms a structured questionnaire was designed. A telephone survey was conducted at 3, 6, and 12 months after discharge to monitor the progression of symptoms over time (Fig. 1).

To safeguard the privacy of patients, all data and biological samples gathered were pseudonymized, and they were identified solely through the assigned barcode. Data collection took place within a database established on the REDCap platform (www.project-redcap.org), based at Harvard Catalyst, Boston, USA, in strict compliance with prevailing GDPR legislation. A data protection impact assessment was implemented.

All procedures adhered to ethical standards set forth by the responsible committee on human experimentation and were in accordance with the Helsinki Declaration of 1975, as amended in 2000. The study received approval from the Ethics Committee (name of the Approval Committee: Milano Area 1, protocol number 0037072). All participants or their responsible guardians were asked for and gave their written consent after being informed about the nature of the study.

### Methods

#### Collection of saliva swabs

Upon admission, saliva swabs were collected from all patients using Lollisponge (LolliSponge™, Copan, Brescia, Italy) [22], within 24 h of admission. The LolliSponge™ was placed in the mouth for one minute to allow the sponge to absorb saliva. Samples, which are self-preservative due to the nature of saliva, were stored at room temperature (RT) without transport medium and were subjected to testing for SARS-CoV-2 using quantitative reverse transcription polymerase chain reaction (qRT-PCR) assays.

Viral RNA was manually extracted utilizing the QIAamp Viral RNA Mini Kit (QIAGEN, Hilden, Germany). RT-PCR genotyping assays were conducted using the COVID-19 Ultra Variant Catcher (Clonit Srl., Milan, Italy), the Allplex SARS-CoV-2 Variants (Arrow Diagnostics Srl., Genoa, Italy), or the multiplexed RT-qPCR developed by the English consortium (https://www.protocols.io/view/multiplexed-rt-qpcr-to-screenfor-sars-cov-2-b-1-1-br9vm966?version_warning=no). For samples with a cycle threshold (Ct) < 30, full genome sequences were acquired through a modified version of the Artic Protocol (https://artic.network/ncov-2019) using the Illumina DNA Prep and the IDT ILMN DNA/RNA Index kit (Illumina, San Diego, CA, USA). Sequencing was conducted on the Illumina Miseq platform utilizing a 2 × 200 cycle paired-end sequencing protocol. Results were aligned and mapped to the reference genome obtained from GISAID (https://www.gisaid.org/, accession ID: EPI_ISL_406800) using Geneious Prime software v. 11.1 (http://www.geneious.com, Biomatters, Auckland, New Zealand). The SARS-CoV-2 lineages and clades were determined using the Pangolin COVID-19 Lineage Assigner v. 4.1.1 (https://pangolin.cog-uk.io/) and Nextclade v. 2.4.1 (https://clades.nextstrain.org).

### Statistical analysis

To summarize the data, categorical variables are presented as counts and percentages, and comparisons between groups were performed using the chi-square test or Fisher’s exact test as appropriate. Quantitative variables are reported as mean and standard deviation (SD) if the empirical distribution shows a symmetric and unimodal pattern and as median and interquartile range (IQR) otherwise.

## Results

Our study included 167 patients, comprising 77/167 (46.1%) females and 90/167 (53.9%) males, with a median age 1 year (IQR 5; range 0–18). Within our cohort, 92 (55.0%) were children under 2 years of age, 50 (30.0%) were aged between 2 and 10 years, and 24 (14.3%) were older than 10 years. None of the patients required intensive care, and chronic comorbidities were present in 49/167 (29.3%) cases. Table 1 provides a detailed overview of the demographic characteristics of the enrolled patients.

**Table 1:** Demographic characteristics of enrolled patients. NA = Not Available

Features	Number of patients (%) *n* = 167
**Age**	
< 2 y	92 (55.1%)
2–10 y	50 (30.0%)
> 10 y	24 (14.3%)
NA	1 (0.6%)
**Gender**	
Female	77 (46.1%)
Male	90 (53.9%)
**Ethnic group**	
Arab	18 (10.8%)
Asian	10 (6.0%)
Latin American	17 (10.2%)
Caucasian	122 (73.1%)
NA	0 (0.0%)
Comorbidities	
-Diabetes	4 (2.4%)
-Obesity	1 (0.6%)
-Chronic malnutrition	1 (0.6%)
-Cardiac congenital malformations	2 (1.2%)
-Asthma	1 (0.6%)
-Chronic_Kidney_Disease	2 (1.2%)
-Gastrointestinal and/or liver disorders	5 (3.0%)
-Neurological_and/or neuropsychiatric disorders	16 (9.5%)
-Immunological_disorder	1 (0.6%)
-Malignant_neoplasm	2 (1.2%)
-Genetic_syndrome	4 (2.4%)
-Others	24 (14.4%)

### Data upon admission

Among the hospitalized children, 70 (41.9%) had a documented history of contact with a SARS-CoV-2 positive individual. Notably, mothers (42.9%) and fathers (29.6%) were the most common sources of contact for our patients.

Upon hospital admission, 9 patients (5.4%) presented as asymptomatic, with SARS-CoV-2 infection incidentally discovered during routine surveillance nasal swabs conducted for non-COVID-related hospitalizations.

Among symptomatic children, the majority (138; 87.3%) were admitted due to a history of fever, often accompanied by cough in 63 (39.9%) of cases, runny nose in 44 (27.8%) of cases, wheezing in 9 (5.7%) of cases, and fatigue or dyspnea in 34 (21.5%) of cases. Additionally, hypoalimentation was reported in 41.9% of patients, while gastrointestinal symptoms, including abdominal pain, vomiting, and diarrhea, were observed in 58 (34.7%) of patients. Neurological symptoms, such as seizures and altered consciousness, were noted in 17 (10.2%) of cases. Symptoms like muscle aches, joint pain, conjunctivitis, skin rashes, and lymphadenopathy were infrequently reported (see Table 2).

**Table 2:** Admission signs and symptoms of enrolled patients according to age groups. * 1 patient not assigned to any of the age classes, due to missing value

Symptom	< 2 years (*n* = 92)	2–10 years (*n* = 50)	> 10 years (*n* = 24)	Total (*n* = 167)^*^
**General symptoms**				
Hystory of fever	78 (85.9%)	39 (78.0%)	19 (79.2%)	138 (82.6%)
**Respiratory symptoms**				
Cough	37 (40.2%)	16 (32.0%)	10 (41.7%)	63 (37.7%)
Runny nose (rhinorrhoea)	31 (33.7%)	9 (18.0%)	4 (16.7%)	44 (26.3%)
Ear pain	1 (1.1%)	0 (0.0%)	0 (0%)	1 (0.6%)
Wheezing	4 (4.3%)	2 (4.0%)	3 (12.5%)	9 (3.6%)
Shortness of breath (dyspnea)	8 (8.7%)	7 (14.0%)	9 (37.5%)	24 (14.4%)
**Muscoloskeletal symptoms**				
Muscle aches (myalgia)	0 (0.0%)	4 (8.0%)	4 (16.7%)	8 (4.9%)
Joint pain (arthralgia)	0 (0.0%)	3 (6.0%)	2 (8.3%)	5 (3.0%)
Fatigue / malaise	2 (2.2%)	6 (12.0%)	6 (25.0%)	14 (8.4%)
**Neurological symptoms**				
Headache	0 (0%)	7 (14.0%)	5 (20.8%)	12 (7.1%)
Alteredconsciousness/confusion	2 (2.2%)	6 (12.0%)	2 (8.3%)	10 (6.0%)
Seizures	4 (4.3%)	13 (26.2%)	1 (4.2%)	18 (10.8%)
**Gastrointestinal symptoms**				
Abdominal pain	2 (2.2%)	9 (18.0%)	6 (25.0%)	17 (10.2%)
Vomiting / nausea	20 (21.7%)	13 (26.0%)	8 (33.3%)	42 (25.1%)
Diarrhoea	15 (16.3%)	2 (4.0%)	5 (20.8%)	22 (13.2%)
Hypoalimentation	44 (47.8%)	18 (36.0%)	8 (33.3%)	70 (41.9%)
**Skin mucosal symptoms**				
Conjunctivitis	4 (4.3%)	2 (4.0%)	0 (0.0%)	6 (3.6%)
Skin rash	3 (3.3%)	6 (12.0%)	1 (4.2%)	10 (6.0%)
Lymphadenopathy	0 (0%)	6 (12.0%)	1 (4.2%)	7 (4.2%)

It is worth noting that vomiting, diarrhea, and hypoalimentation appeared to be more prevalent among children under 2 years of age, while neurological symptoms were more common in children aged 2–10 years and adolescents.

Table 2 summarizes the signs and symptoms observed upon admission in the enrolled patients, categorized according to different age groups. Statistical significance was reached for: shortness of breath (*p* = 0.0039), all muscoskeletal symptoms (*p* = 0.0006, 0.01169, and 0.0005 for myalgia, arthralgia and fatigue, respectively), all neurological symptoms (*p* < 0.0001, 0.0406, 0.0004 for headache, altered consciousness and seizures, respectively), abdominal pain (*p* = 0.0002), diarrhea (*p* = 0.0389) and lymphadenopathy (*p* = 0.0019).

Regarding patients for whom it was feasible to determine the SARS-CoV-2 variant via saliva swab (*n* = 51), the Delta variant was identified in 11 children (21.6%). These cases were all hospitalized between November 2021 and December 2021, with the exception of one patient admitted in January 2022. Specifically, all of these subjects carried Delta descendant variants within the 21 J clade. In contrast, the Omicron variant was detected in the remaining 40 patients (78.4%), all of whom were hospitalized from January 2022 to June 2023. Among these, 27.5% (*n* = 11) had the BA.1 variant and its sublineages (clade 21 K), 15% (*n* = 6) carried BA.2 (clade 21 L), and 57.5% (*n* = 23) had the BA.5 variant (clade 22B). Additionally, three cases of XBB recombinants were observed (7.5%) between March and May 2023.

As shown in Table 3, while neurological symptoms appeared to be more prevalent in cases with the Omicron variant, and gastrointestinal symptoms were seemingly more common in Delta variant cases, it’s important to note that none of these symptoms reached statistical significance.

**Table 3:** Distribution of symptoms between Delta variant, Omicron variant and non-detectable variant (ND)

Symptoms	Delta (*n* = 11)	Omicron (*n* = 40)	ND (*n* = 62)	Total (*n* = 113)
**General symptoms**				
Hystory of fever	10 (90.9%)	36 (90%)	48 (77.4%)	94 (83.2%)
**Respiratory symptoms**				
Cough	6 (54.5%)	18 (45%)	22 (35.4%)	46 (40.7%)
Runnynose (Rhinorrhoea)	5 (45.4%)	8 (2%)	18 (29.0%)	31 (27.4%)
Earpain	1 (9.0%)	0 (0%)	0 (0%)	1 (0.9%)
Wheezing	2 (18.1%)	1 (0.2%)	4 (6.4%)	7 (6.2%)
Shortness of breath (Dyspnea)	2 (18.1%)	3 (0.7%)	9 (14.5%)	14 (12.4%)
**Muscoloskeletal symptoms**				
Muscle aches (Myalgia)	0 (0%)	0 (0%)	6 (9.8%)	6 (5.3%)
Joint pain (Arthralgia)	1 (9.0%)	0 (0%)	3 (4.8%)	4 (3.5%)
Fatigue / Malaise	1 (9.0%)	1 (0.2%)	10 (16.1%)	12 (10.6%)
**Neurological symptoms**				
Headache	1 (9.0%)	3 (0.7%)	4 (6.5%)	8 (7.0%)
Altered consciousness/confusion	0 (0%)	1 (2.5%)	6 (9.8%)	7 (6.2%)
Seizures	0 (0%)	4 (10%)	5 (8.0%)	9 (8.0%)
**Gastrointestinal symptoms**				
Abdominal pain	1 (9.0%)	1 (2.5%)	8 (12.9%)	10 (8.8%)
Vomiting / Nausea	5 (45.4%)	10 (25%)	18 (29.0%)	33 (29.2%)
Diarrhoea	2 (18.1%)	4 (15%)	9 (14.5%)	15 (13.2%)
Hypoalimentation	6 (54.5%)	15 (37.5%)	33 (53.2%)	54 (47.8%)
**Skinmucosal symptoms**				
Conjunctivitis	0 (0%)	1 (2.5%)	2 (3.2%)	3 (2.7%)
Skin rash	1 (9.0%)	2 (5%)	5 (8.0%)	8 (7.0%)
Lymphadenopathy	0 (0%)	2 (5.4%)	3 (4.8%)	5 (4.4%)

Out of the 167 patients enrolled, 134 (80.2%) had not received the SARS-CoV-2 vaccination prior to their infection (19 subjects with unknown or not assessed status). Among them, a significant majority (71.6%) were ineligible for vaccination due to their age (5 subjects with not available information).

### Monitoring for long COVID

**Fig. 2:**
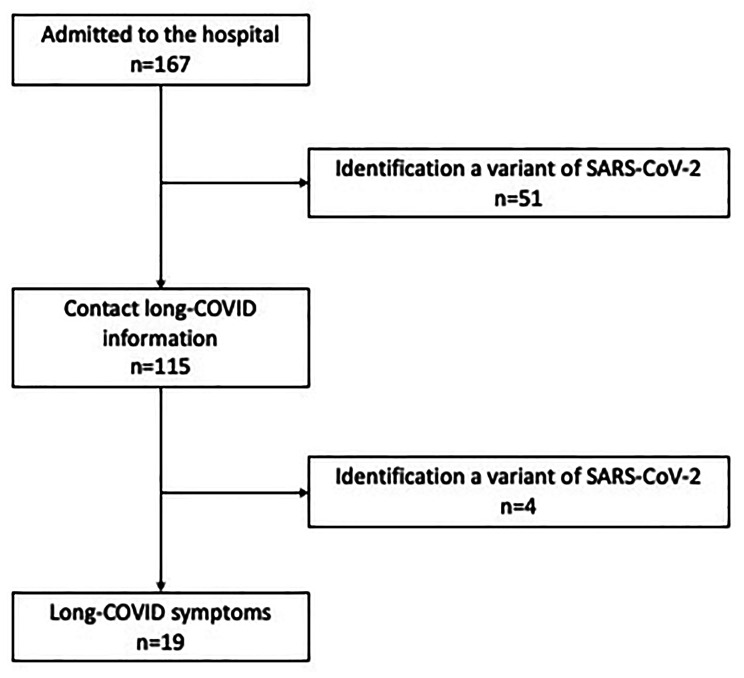
Flowchart illustrating the patients’ medical history

In Fig. 2, we present a flowchart depicting the patients’ medical history.

Out of the 115 patients (55 females and 60 males) who were followed up for at least 6 months after their discharge (comprising 67/115 patients or 58.3% aged < 2 years, 33/115 or 28.7% aged between 2 and 10 years, and 15/115 or 13.0% aged older than 10 years), 19 patients (16.5%, including 7 females and 12 males) reported experiencing at least one symptom indicative of Long COVID, with most of them presenting a combination of symptoms. The patients who developed Long COVID symptoms did not have underlying health conditions, except for one case of diabetes. All symptoms were reported at 3 months from discharge and lasted at least two months.

The most commonly reported symptoms included weight loss (6/19, 31.6%), inappetence (5/19, 26.3%), chronic cough (4/19, 21.1%), fatigue (4/19, 21.1%). and sleep disturbances, wheezing, abdominal pain and mood disorders (3/19, 15.8%). Additionally, patients reported symptoms such as headache, cognitive changes, muscle aches, joint pain or swelling, (see Table 4). Table 4 summarizes the Long COVID symptoms according to different age groups.

**Table 4:** Symptoms of long COVID according to age groups

Symptom	< 2 years (*n* = 7)	2–10 years (*n* = 5)	> 10 years (*n* = 7)	Total (*n* = 19)
Weight loss	2 (28.6%)	3 (60.0%)	1 (14.3%)	6 (31.6%)
Inappetence	2 (28.6%)	2 (40.0%)	1 (14.3%)	5 (26.3%)
Fatigue	0 (0,0%)	1 (20.0%)	3 (42.8%)	4 (21.1%)
Sleep disorders	3 (42.8%)	0 (0.0%)	1 (14.3%)	4 (21.1%)
Chronic cough	0 (0.0%)	2 (40.0%)	2 (28.6%)	4 (21.1%)
Wheezing	1 (14.3%)	1 (10.0%)	0 (0.0%)	2 (10.5%)
Abdominal pain	0 (0.0%)	1 (33.3%)	1 (14.3%)	2 (10.5%)
Mood disorders	0 (0.0%)	0 (0.0%)	2 (28.6%)	2 (10.5%)
Headache	0 (0.0%)	0 (0.0%)	1 (14.3%)	1 (5.3%)
Cognitive alterations	1 (0.0%)	0 (0.0%)	0 (0.0%)	1 (5.3%)
Muscle aches	0 (0.0%)	1 (0.0%)	1 (14.3%)	2 (10.5%)
Joint pain or swelling	0 (0.0%)	1 (100%)	0 (0.0%)	1 (10.5%)
Tachycardia	0 (0.0%)	0 (0.0%)	0 (0.0%)	0 (0.0%)

As depicted in Table 4, fatigue, chronic cough, as well as cognitive and psychological issues like concentration difficulties, mood disorders, and eating disorders were predominantly observed in adolescents. On the other hand, infants and toddlers exhibited recurrent wheezing, failure to thrive, and sleep disturbances more frequently.

Of the 19 patients who reported symptoms indicative of Long COVID, only 3 could be identified with a specific SARS-CoV-2 variant through saliva swabs. Among these cases, 2 were associated with the Omicron variant (2 BA.1 and 1 BA.2), and 1 was linked to the Delta variant.

For 18 out of the 19 patients (94.7%), the Long COVID symptoms necessitated consultation with a medical professional, while 11 out of 19 (57.9%) required medication, and 2 out of 19 (10.5%) needed hospitalization. The hospitalizations were prompted by a range of issues, including eating disorders, wheezing, and a combination of weight loss and joint pain.

Among preschool-aged children, the majority (7 out of 10, 70%) experienced frequent limitations in their daily activities at home, which placed a substantial burden on their families. For all school-aged children in the study (*n* = 9), they faced limitations in their daily lives, both at home and during school activities. Furthermore, in 6 out of these 9 cases (66.6%), Long COVID symptoms interfered with friendships, leisure activities, and physical activities, resulting in high levels of stress within their families.

Patients aged 7 years and older (*n* = 7) were asked to rate their quality of life during the period in which they experienced Long COVID symptoms on a scale from 0 (the worst) to 10 (the best). The responses ranged from 0/10 to 8/10, with an average rating of 4.

## Discussion

While the definition of long COVID has been established in the literature, there remains a dearth of data on its epidemiology and clinical presentation, particularly in the pediatric population [23]. This study sheds light on the demographic and clinical characteristics of a cohort of hospitalized pediatric patients with SARS-CoV-2 infection, focusing on the development of symptoms associated with long COVID after discharge. Notably, we observed a long-COVID prevalence of 13% in our population.

Our findings align with previous reports indicating that most children hospitalized for COVID-19 are infants or toddlers. This observation is consistent with multicenter studies highlighting the two most susceptible age groups for hospitalization in 2021 as 0–4 years and 12–17 years [24]. The high rate of hospitalization among younger children may be attributed, at least in part, to the widespread circulation of the highly transmissible Delta variant [25]. It’s worth noting that the majority of patients in our cohort had not received the SARS-CoV-2 vaccination before infection, primarily due to age restrictions on vaccination eligibility. The availability of COVID-19 vaccines for children aged 5 years and older in Lombardy, Italy, was only extended until December 27, 2022, and was further expanded to include the age group of 6 months to 4 years. Additionally, the lower hospitalization rate among adolescents may be attributed to the availability of COVID-19 vaccines for individuals over 12 years of age since June 2021 in Lombardy, Italy, underscoring the substantial effectiveness of vaccines in preventing severe COVID-19 in this age group [25].

At hospital admission, the most frequently reported symptoms in our pediatric cohort were fever, respiratory symptoms, hypoalimentation, gastrointestinal symptoms (including abdominal pain, vomiting, and diarrhea), and neurological symptoms (including seizures and altered consciousness). Importantly, these symptoms show significant differences based on age. The majority of patients had documented contact with a SARS-CoV-2 positive individual, most commonly one of their parents, which is consistent with data from other Italian pediatric cohorts [26]. This underscores the high incidence of infections within close contacts and familial clusters.

According to the clinical definition proposed by the WHO, Long COVID, also known as Post-Acute Sequelae of SARS-CoV-2 Infection, is defined as the persistence or onset of symptoms within 3 months of SARS-CoV-2 infection, lasting for at least 2 months without any alternative explanation [1]. The reported prevalence of long COVID symptoms varies widely worldwide [27]. According to a recent systematic review, the prevalence of long-COVID in children and adolescents was 25.24% [23]. Bloise et al. reported that the pediatric Italian cohort including both hospitalized children and children whose disease was managed at home, the prevalence of long-lasting symptoms was around 20% [26].

In our cohort, the prevalence of long COVID was lower (16.5%) compared to literature data [23]. This discrepancy may be partially due to the age distribution of our population, with a higher prevalence in infants, who may not manifest certain symptoms commonly associated with long COVID, such as mood symptoms and fatigue [23].

Similar to previous studies on pediatric cohorts, we found no statistically significant difference in the prevalence of long COVID symptoms between males and females. Unlike studies in adults where female gender is cited as a risk factor for long COVID [28, 29], gender-related differences in the prevalence of persistent symptoms in children remain inconclusive [23, 30].

The most prevalent long COVID symptoms in our cohort were weight loss 31.6%, inappetence 26.3%, chronic cough 21.1%, fatigue 21.1%, and sleep disturbances, wheezing, abdominal pain and mood disorders 15.8% [23].

Mental health issues are also noteworthy in children with prior SARS-CoV-2 infection, with symptoms such as anxiety, depression, sleep disturbances, appetite changes, and impaired social interactions being frequently reported [31]. These conditions may be partly attributed to the broader impact of the pandemic on the lives and relationships of children and adolescents. However, there may be underlying functional pathophysiology contributing to these symptoms, as children with long COVID have been found to exhibit brain hypometabolism patterns similar to those seen in adults with long COVID [32]. In line with existing literature findings [23], our cohort also exhibited frequent occurrences of fatigue, chronic cough, concentration difficulties, mood disorders, eating disorders, sleep disturbances, and respiratory symptoms, without any significant differences among different age groups.

In adults, it is well-established that long COVID symptoms can have a detrimental impact on individuals’ functioning and overall quality of life [33]. To the best of our knowledge, previous studies have not delved into how these symptoms affect the daily activities of children and adolescents. In our study, we included a dedicated segment in our telephone survey to assess patients’ limitations in their daily lives and their own evaluations of their quality of life. In accordance with findings from adult cohorts, we observed a notable decline in all aspects of daily life, particularly at home and in school. This had a considerable burden on the families of the patients, and school-aged children reported a diminished self-assessment of their quality of life.

These findings underscore the necessity for rehabilitation interventions in these patients, particularly targeting symptoms such as dyspnea, chronic cough, and fatigue, with the aim of achieving functional improvement, as is recommended for adults [34].

This study has some limitations. Firstly, in nearly 69.5% of cases, it was not possible to identify VOCs through saliva swabs due to negative results or high cycle threshold (Ct) values, limiting our ability to detect statistically significant differences in long-term and short-term symptoms between patients with SARS-CoV-2 Delta or Omicron variants. The issue of viral load determination is in line with recent data suggesting that viral load in nasopharyngeal swabs is higher than in salivary specimens, especially after symptoms onset and irrespective of food or beverage consumption [35]. Furthermore, there were cases where salivary samples were collected with delays due to logistical reasons, such as stays in the emergency room or at other facilities before admission to the Buzzi hospital. Additionally, since intensive care was not required for any patient in our population, we could not compare the prevalence of long COVID based on the severity of the acute phase of infection. Finally, a control group of hospitalized children not experiencing COVID-19 was not included.

## Conclusions

This study underscores that long COVID is a significant concern in the pediatric population across all age groups and does not exhibit a gender-specific prevalence difference. Our data reinforce the importance of continuously monitoring the impact of long-COVID in infants, children, and adolescents. A follow-up following SARS-CoV-2 infection is therefore advisable, with symptom investigation tailored to the patient’s age. Further studies are warranted to detail the correlation between VOCs and symptoms related to long COVID.

### Electronic supplementary material

Below is the link to the electronic supplementary material.

Supplementary Material 1

## Data Availability

All data are reported in the paper.

## Abbreviations

IQR: Interquartile range
qRT-PCR: Quantitative reverse transcription polymerase chain reaction
RT: Room temperature
SARS: Severe acute respiratory syndrome
SD: Standard deviation
VOCs: Variants of concern
