# Supplementary material for: Long-term effects of SARS-CoV-2 infection in hospitalized children: findings from an Italian single-center study
Source: Ital J Pediatr. 2024 Feb 14;50:27. doi: 10.1186/s13052-024-01596-y (PMC10865522; doi:10.1186/s13052-024-01596-y)
Supplement: Supplementary file 1 — Supplementary Material 1 [file 13052_2024_1596_MOESM1_ESM.doc]

Milano, 28 December 2023

Dear Prof. Giovanni Corsello,

With pleasure, we resubmit the revised version of our manuscript “Long-Term Effects of SARS-CoV-2 Infection in Hospitalized Children: Findings from an Italian Single-Center Study**”** by Calcaterra et al. to be taken into consideration for publication in the Italian Journal of Pediatrics.

A point-by-point response to the reviewer’s comments is provided below (the reviewer’s comments are in bold and our answers are in red plain text).

We wish to thank the reviewers for their positive feedback and constructive comments and we hope that the manuscript is now suitable for publication.

Thank you for your kind attention and consideration.

Yours Sincerely,

Valeria Calcaterra

**Reviewer #2:**

Thank for your offer

Firstly, your article studied the common symptoms and Long COVID manifestations of children with COVID-19 infections between different ages and different sub-groups. The follow-up data does indicate that symptoms will persist. Secondly, I think the shortcoming of this article is that it did not mention whether the children involved in the study had  foundational diseases.

R: we thank the reviewer for your positive comment. The comorbidities of all patients at the hospitalization are reported in table 1. We specified in the text, that the patients who developed long COVID symptoms did not have underlying health conditions, except for one case of diabetes.
